# Neonatal sepsis definitions from randomised clinical trials

**DOI:** 10.1038/s41390-021-01749-3

**Published:** 2021-11-06

**Authors:** Rían Hayes, Jack Hartnett, Gergana Semova, Cian Murray, Katherine Murphy, Leah Carroll, Helena Plapp, Louise Hession, Jonathan O’Toole, Danielle McCollum, Edna Roche, Elinor Jenkins, David Mockler, Tim Hurley, Matthew McGovern, John Allen, Judith Meehan, Frans B. Plötz, Tobias Strunk, Willem P. de Boode, Richard Polin, James L. Wynn, Marina Degtyareva, Helmut Küster, Jan Janota, Eric Giannoni, Luregn J. Schlapbach, Fleur M. Keij, Irwin K. M. Reiss, Joseph Bliss, Joyce M. Koenig, Mark A. Turner, Christopher Gale, Eleanor J. Molloy

**Affiliations:** 1 Discipline of Paediatrics, Trinity College Dublin, the University of Dublin & Children’s Hospital Ireland (CHI) at Tallaght, Dublin, Ireland; 2 John Stearne Medical Library, Trinity College Dublin, St. James’ Hospital, Dublin, Ireland; 3 Trinity Translational Medicine Institute, St James Hospital, Dublin, Ireland; 4 Trinity Research in Childhood Centre (TRiCC), Trinity College Dublin, Dublin, Ireland; 5 Department of Paediatrics, Tergooi Hospital, Blaricum, The Netherlands; 6 Department of Paediatrics, Amsterdam UMC, University of Amsterdam, Emma Children’s Hospital, Amsterdam, The Netherlands; 7 Neonatal Health and Development, Telethon Kids Institute, Perth, WA Australia; 8 Neonatal Directorate, King Edward Memorial Hospital for Women, Perth, WA Australia; 9 Radboud Institute for Health Sciences, Department of Neonatology, Radboud University Medical Center, Amalia Children’s Hospital, Nijmegen, The Netherlands; 10 Division of Neonatal-Perinatal Medicine, Department of Pediatrics, Columbia University Medical Center, New York City, NY USA; 11 Department of Pediatrics, University of Florida, Gainesville, FL USA; 12 Department of Pathology, Immunology, and Laboratory Medicine, University of Florida, Gainesville, FL USA; 13 Department of Neonatology, Pirogov Russian National Research Medical University, Moscow, Russia; 14 Neonatology, Clinic for Paediatric Cardiology, Intensive Care and Neonatology, University Medical Centre Göttingen, Göttingen, Germany; 15 Neonatal Unit, Department of Obstetrics and Gynecology, Motol University Hospital and Second Faculty of Medicine, Prague, Czech Republic; 16 Institute of Pathological Physiology, First Faculty of Medicine, Charles University, Prague, Czech Republic; 17 Clinic of Neonatology, Department Mother-Woman-Child, Lausanne University Hospital and University of Lausanne, Lausanne, Switzerland; 18 Paediatric Critical Care Research Group, Child Health Research Centre, University of Queensland, Brisbane, Australia; 19 Paediatric Intensive Care Unit, Queensland Children’s Hospital, Brisbane, Australia; 20 Department of Pediatrics, Bern University Hospital, University of Bern, Bern, Switzerland; 21 Department of Pediatrics, Division of Neonatology, Erasmus MC-Sophia Children’s Hospital, Rotterdam, The Netherlands; 22 Department of Pediatrics, Women & Infants Hospital of Rhode Island, Alpert Medical School of Brown University, Providence, USA; 23 Division of Neonatology, Saint Louis University, Edward Doisy Research Center, St. Louis, MO USA; 24 Institute of Translational Medicine, University of Liverpool, Centre for Women’s Health Research, Liverpool Women’s Hospital, Liverpool, UK; 25 Neonatal Medicine, School of Public Health, Faculty of Medicine, Chelsea and Westminster campus, Imperial College London, London, UK; 26 Paediatrics, Coombe Women’s and Infant’s University Hospital, Dublin, Ireland; 27 Neonatology, CHI at Crumlin, Dublin, Ireland

## Abstract

**Introduction:**

Neonatal sepsis is a leading cause of infant mortality worldwide with non-specific and varied presentation. We aimed to catalogue the current definitions of neonatal sepsis in published randomised controlled trials (RCTs).

**Method:**

A systematic search of the Embase and Cochrane databases was performed for RCTs which explicitly stated a definition for neonatal sepsis. Definitions were sub-divided into five primary criteria for infection (culture, laboratory findings, clinical signs, radiological evidence and risk factors) and stratified by qualifiers (early/late-onset and likelihood of sepsis).

**Results:**

Of 668 papers screened, 80 RCTs were included and 128 individual definitions identified. The single most common definition was neonatal sepsis defined by blood culture alone (*n* = 35), followed by culture and clinical signs (*n* = 29), and then laboratory tests/clinical signs (*n* = 25). Blood culture featured in 83 definitions, laboratory testing featured in 48 definitions while clinical signs and radiology featured in 80 and 8 definitions, respectively.

**Discussion:**

A diverse range of definitions of neonatal sepsis are used and based on microbiological culture, laboratory tests and clinical signs in contrast to adult and paediatric sepsis which use organ dysfunction. An international consensus-based definition of neonatal sepsis could allow meta-analysis and translate results to improve outcomes.

## Introduction

An estimated 3,000,000 newborns are affected by sepsis annually^1^ with mortality set to reach 375,000 in 2019 (ref. ^2^). Despite ongoing advances in molecular diagnostics,^3^ the accurate and timely diagnosis of sepsis in neonates remains challenging. Current conventional gold standard diagnosis of sepsis based on microbial culture does not appear to reliably rule-out sepsis, with reported rates of ‘culture-negative’ or ‘suspected’ sepsis varying widely in the literature. While some experts advocate to consider sepsis evaluations completed after 48–72 h of negative blood cultures^4^^,^^5^ data available from two large randomised controlled trials (RCTs) in recent years (INIS^6^ and ELFIN^7^) show culture-negative sepsis rates of 56% and 46%, respectively.

Antibiotic overuse and resistance are increasingly common but under-recognition of sepsis remains an issue resulting in campaigns from the World Health Organisation (WHO).^8^

Sepsis-3 (ref. ^9^), the recent consensus definitions for sepsis and septic shock in adults, primarily uses multiorgan dysfunction rather than microbial culture in the screening and diagnosis of sepsis. Even when microbiological tests are completed, culture-positive ‘sepsis’ is observed in only 30–40% of cases in adults.^9^ Neonatal sepsis literature is therefore at variance with adult and paediatric sepsis consensus and a greater emphasis on multiorgan dysfunction may be merited. The neonatal Sequential Organ Failure Assessment Score (nSOFA)^10^ characterises neonatal organ dysfunction and predicts mortality in this setting, and thus the potential for its use in the definition of neonatal sepsis. Measures of neonatal organ dysfunction have been developed but remain distinct from definitions of infection or sepsis at present.

Neonatal sepsis, especially in vulnerable (e.g. very low birth weight) populations, is strongly associated with increased rates of complications of prematurity^11,12^ and adverse neurodevelopmental outcomes.^13–18^ These unfavourable outcomes emphasise the need for rapid recognition of sepsis and initiation of antibiotic therapy. However, unnecessary empiric antibiotic prescription is associated with increasing long term morbidity and mortality,^19–28^ and current antibiotic prescribing habits have been shown to vary widely internationally.^29–32^ These issues emphasise the need for a consensus definition of neonatal sepsis so that standardised outcomes data can feed back into diagnostic and therapeutic improvement.^33^

In this study we aimed to systematically review the definitions of neonatal sepsis used in published RCTs. This process is in tandem with a separate analysis^34^ which examines the use of definitions of neonatal sepsis in observational and experimental studies from neonatal surveillance and research networks. The goal here is to identify commonalities and differences between definitions of neonatal sepsis in order to define core themes for Delphi and consensus processes to develop a universally accepted definition for neonatal sepsis.

## Methods

### Search strategy

In May 2019, the Embase and Cochrane databases were searched with the algorithm (‘neonatal’ OR ‘newborn’) AND (‘sepsis’ OR ‘septicaemia’). No date restrictions were imposed on results. Only randomised clinical trials in the English language which explicitly stated a definition for neonatal sepsis were included. All other publications, including observational studies, reviews and RCTs for which a full text was not available, and RCTs that included non-neonatal paediatric patients were excluded.

### Data collection and analysis

The following data were extracted from the included studies: year of publication, sample size, verbatim definition of sepsis, and primary and secondary outcomes measured. After a preliminary review of the data, the authors characterised five primary criteria from which all the definitions of neonatal sepsis were composed: microbiological culture, clinical signs of infection, laboratory signs of infection, radiological signs of infection, and the presence of risk factors for infection. Many RCTs presented algorithmic definitions of neonatal sepsis, e.g. ‘Neonatal sepsis was defined as a positive microbial culture plus either laboratory signs of sepsis or clinical signs of sepsis’. To enable adequate analysis, these algorithms were broken down by their component primary criteria. For instance, in the above definition that diagnosed neonatal sepsis as ‘positive culture plus clinical signs or laboratory signs of infection’ we took this as two separate definitions of neonatal sepsis: (1) culture plus clinical signs of infection and (2) culture plus laboratory signs of infection. For each primary criterion, a detailed breakdown of the relevant secondary criteria was collected. Where definitions included microbiological culture as a criterion, the collected secondary criteria were: culture site(s), the number of samples required to be positive, pathogens cultured for, the time the sample was taken, and the incubation time. For laboratory signs, clinical signs, radiological signs, and risk factors for infection, the frequency of individual signs was recorded. Where definitions specified a required number of positive signs in a specific category, these figures were recorded. Many definitions of neonatal sepsis included qualifiers (e.g. early/late, suspected/proven, coagulase-negative staphylococci (CoNS) and additional criteria for severe sepsis/septic shock). These qualifiers were recorded and a sub-analysis of the criteria they used was performed.

## Results

### Search results

**Fig. 1:**
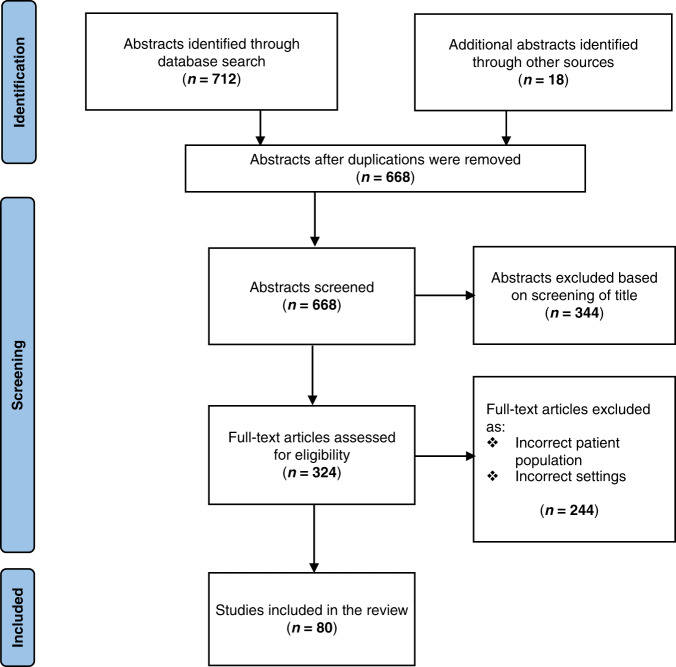
PRISMA flow diagram of studies included in the systematic review of definitions of neonatal sepsis in randomised controlled trials.

A total of 668 papers were identified by the search, of which 344 were excluded based on screening of the title and abstract; 80 of the remaining 324 studies met the inclusion criteria for the review as RCTs and were included in the analysis of the full text. The selection of studies was undertaken in accordance with the PRISMA (Preferred Reporting Items for Systematic Reviews and Meta-Analyses) guidelines (Fig. 1).

### Study characteristics

A total of 80 randomised clinical trials^4,5,8,35–122^ were included in the review from 1986 to 2019 yielding a total of 128 individual definitions of neonatal sepsis when broken down by component primary criteria (Table 1): microbiological culture, clinical signs of infection, laboratory signs of infection, radiological signs of infection, and the presence of risk factors for infection. The tabulated raw dataset is available in Appendix 1. The total sample size comprised by the trials was 40,992 neonates, the median sample size was 150, and the mean sample size was 512. The most represented primary criterion was microbiological culture, which was a component of 83 separate definitions. The frequency of other primary criteria appeared as follows: clinical signs of infection (*n* = 81), biochemical/haematological signs of infection (*n* = 48), radiological signs of infection (*n* = 8), risk factors for infection (*n* = 2). None of the included definitions were consensus or guideline definitions.

**Table 1:** Definitions of neonatal sepsis by primary criteria.

Combination of primary criteria	*N*
Culture alone	35
Culture + signs	29
Signs + laboratory	25
Culture + signs + laboratory	12
Signs alone	7
Culture + labs	6
Signs + radiology	6
Laboratory alone	4
Signs + risk factors	2
Culture + laboratory + radiology	1
Radiology alone	1

### Primary outcomes

1. Microbiological culture
  Microbiological culture was a component of 83 out of 128 definitions of neonatal sepsis (Table 1). These 83 definitions were from 68 different papers.
  *Culture source***:** Of the 83 culture-related definitions, 74 specified a site the culture sample was taken from, while 9 mentioned ‘culture’ without specifying a sample site. Of the 74 that specified culture sites, the frequency of each site mentioned is outlined in Table 2. Three definitions required two positive cultures to diagnose sepsis.

**Table 2:** Definitions mentioning specific culture source.

Culture source	*N*
Blood	71
Cerebrospinal fluid	29
Urine	10
Skin/surface	4
Pus [unspecified]	3
Tracheal aspirate	2
Synovial fluid	1
Peritoneal fluid	1
Intravascular catheter	1
Any sterile site	1
  *Pathogen***:** 12 definitions specified a pathogen sought by microbiological culture. Five specified bacteria, 4 specified fungi, and the remainder mentioned ‘known virulent pathogens’, or ‘not *Staphylococcus epidermidis*’.
  *Incubation time*: Only one study mentioned incubation time, which outlined that a microorganism was regarded as infectious if it grew within 48 h incubation.
2. Clinical signs of infection
  Clinical signs were a component of 81 definitions. Seven defined sepsis based on signs alone and 74 defined sepsis based on signs in conjunction with other primary criteria, culture being the most common. These 81 definitions represent 35 RCTs that gave only 1 definition of sepsis that included clinical signs, and a further 18 papers which had multiple definitions that included clinical signs. Ten of these 18 papers gave a definition based on culture and clinical signs, and another definition based on laboratory criteria and clinical signs. Several other papers which included clinical signs in their definitions also gave multiple definitions that distinguished between possible and probable sepsis. Out of 81 definitions of sepsis to include clinical signs, 41 specified the signs that were looked for (either examples given, or a prescriptive list), the remaining 40 definitions mentioned signs without specifying what signs were looked for 20 definitions explicitly stated how many signs had to be present, with a breakdown as follows: 1 sign (*n* = 8), 2 signs (*n* = 11), and 3 signs (*n* = 1). To categorise the wide variety of clinical signs mentioned, signs were grouped by system, except for ‘requirement for antibiotics’, which was grouped alone.
  *Antibiotic requirement***:** Seven definitions mentioned a ‘requirement for antibiotics’ as a sign of neonatal sepsis. The date range for these seven definitions is 2000–2018. Six of the seven definitions mention a requirement for a 5-day course of antibiotics, intention to treat for 5 days, or neonatal death before completion of a 5-day antibiotic course.
  *Overview of clinical signs***:** The categories of clinical signs appeared in the following order of frequency: systemic (*n* = 32), respiratory (*n* = 29), cardiovascular (*n* = 28), gastrointestinal signs (*n* = 12), neurological (*n* = 12), and miscellaneous (*n* = 8). The frequency of individual signs within each category is outlined in Table 3.

**Table 3:** Signs and symptoms present and frequency in the RCT definitions reviewed.

Constitutional		Respiratory		Cardiovascular		Neurological		Gastrointestinal		Miscellaneous	
Symptom	*N*	Symptom	*N*	Symptom	*N*	Symptom	*N*	Symptom	*N*	Symptom	*N*
Lethargy	27	Apnoea	22	Haemodynamic instability	8	Altered consciousness	7	Abdominal distension	11	Disseminated haemorrhage	2
Temperature instability	27	Respiratory distress	12	Hypotension	7	Seizure	6	Vomiting	5	Unexplained bleeding	2
Feeding intolerance	17	Tachypnoea	10	Poor perfusion	7	Hypotonia	4	Hepatomegaly	5	Petechiae	1
Glucose intolerance	9	Ventilatory support	6	Tachycardia	6	Reduced reflexes	2	Splenomegaly	4	Purpura	1
Irritability	5	Supplemental O_2_	6	Bradycardia	6	Bulging fontanelle	1	Jaundice/icterus	4	Pyoderma	1
Hypothermia	4	Desaturations	4	Inotropic/fluid support	5			Increased gastric aspirate	1	Sclerema	1
Hyperthermia	3			CRT > 3 s	5					Conjunctivitis	1
Fever	3	Grunting	4	Pallor	3					Organ dysfunction [unspecified]	3
Poor feeding	3	Cyanosis	3	Rate > 2 SD above normal	2					Staff concern	1
Excessive crying	1	Gagging	1	Shock	2						
Poor cry	1	Apnoea	22	Cardiovascular collapse	2						
Colour	1	Respiratory distress	12	BP < 2 SD below normal	2						
		Tachypnoea	10	Rate instability	1						
				Cold extremities	1
  *Systemic signs*: The 32 definitions specifying constitutional signs represent information from 22 separate papers. The constitutional signs were further stratified into five main categories: lethargy, temperature, feeding, cry, and colour (Table 3).
  *Respiratory signs*: The 29 definitions specifying respiratory signs represent information from 20 separate papers. The listed respiratory signs were further stratified into three main categories: hypoxaemic signs, signs of distress, and requirement for support (Table 3).
  *Cardiovascular signs*: The 28 definitions specifying cardiovascular signs represents information from 19 separate papers. The listed cardiovascular signs were divided into three broad categories: heart rate, blood pressure, and indicators of perfusion (Table 3).
  *Neurological signs*: The 12 definitions specifying neurological signs represent information from 10 individual papers. A total of five neurological signs were mentioned (Table 3).
  *Gastrointestinal signs*: A total of six gastrointestinal signs were mentioned (Table 3).
  *Miscellaneous signs*: The eight definitions that specified miscellaneous signs represent information from six individual papers. A total of nine miscellaneous signs were mentioned (Table 3).
3. Laboratory (microbiological/haematological/biochemical) signs of infection
  Haematological/biochemical signs of infection were a component of 48 definitions of neonatal sepsis, 4 of which defined sepsis based on laboratory criteria alone. The remaining 44 defined sepsis based on laboratory criteria in conjunction with other primary criteria (Table 1). The 48 laboratory-related definitions of sepsis came from 41 separate papers. Of the seven papers that provided two definitions, six papers included two definitions in order to distinguish between definite sepsis and probable sepsis. Of these six papers, three defined probable sepsis as laboratory criteria with clinical signs and definite sepsis as culture with laboratory criteria and clinical signs.
  Although there were 48 laboratory-related definitions of sepsis, only 40 specified the laboratory tests required to make the diagnosis, while the remaining 8 mentioned laboratory parameters, without specifying what tests were necessary. There was a wide variation in the number of laboratory parameters included in each definition and of the 40 definitions that specified laboratory tests to be positive, 21 required at least one parameter to be abnormal, 18 requires at least 2 parameters to be abnormal, and 1 required at least 3 laboratory parameters to be abnormal. A total of 20 individual laboratory tests were mentioned by the 48 definitions of neonatal sepsis that included laboratory criteria as a primary criterion. These 20 individual parameters were grouped into five categories: blood count, serum inflammatory markers, metabolic markers, histopathological findings, and markers of pathogenaemia (Table 4).

**Table 4:** Frequency of laboratory signs in the RCT definitions reviewed.

Laboratory signs	*N*
C-reactive protein	30
Not specified	9
>5 mg/L	1
>9 mg/L	1
>10 mg/L	13
>12 mg/L	1
>20 mg/L	4
>60 mg/L	1
White cell count (WCC)	16
I:T ratio	15
Neutrophil count	13
Platelet count	10
Micro-ESR	8
Band cell count	7
Full blood count (FBC)	3
IL-6	3
Glucose	3
Toxic granules in peripheral smear	2
Bacterial antigen	2
TNF-alpha	1
Procalcitonin	1
Lactate	1
pH	1
Histologic diagnosis of pneumonia	1
Cerebrospinal WCC	1
Viral polymerase chain reaction (PCR)	1
CSF Gram stain	1
4. Radiological signs of infection
  There were eight definitions of neonatal sepsis that included radiological signs of infection and only seven specified which radiological signs were sought. All seven definitions that specified signs specified chest X-ray/evidence of pneumonia as the only relevant radiological exam.
5. Risk factors for infection

There were two definitions of neonatal sepsis that included risk factors for infection as a primary diagnostic criterion. The risk factors mentioned are as follows: amnionitis (*n* = 2), premature rupture of membranes (*n* = 2), foul-smelling amniotic fluid (*n* = 1), prolonged rupture of membranes >24 h (*n* = 1), maternal fever (*n* = 1), maternal urinary tract infection (*n* = 1).

### Qualifiers

1. Early- versus late-onset neonatal sepsis
  No definition of neonatal sepsis specified the timeframe for neonatal sepsis as other than birth to 28 days of life. Two studies provided definitions of both early- and late-onset sepsis. A further 11 studies provided definitions of either early or late-onset sepsis. Of the seven studies that defined early-onset sepsis, one study defined early onset as arising < 48 h of life, five studies as < 72 h, and one study as < 5 days of life. Of the eight studies that defined late-onset sepsis, all eight defined late-onset sepsis as arising ≥ 72 h of life.
2. Definite/probable/possible sepsis
  There were 21 studies that provided definitions that discriminated between degrees of certainty of the diagnosis of sepsis. Of utmost certainty were ‘definite sepsis’ or ‘culture-proven sepsis’, next was ‘probable sepsis’ or ‘clinical sepsis’, followed by ‘possible sepsis’ as defined by some studies.
3. Stringency for coagulase-negative Staphylococci (CoNS)
  Six papers included criteria relating to CONS. Of these, two specified that Staph. epidermidis was disregarded if cultured, and the other four papers (three mention CONS, one mentions Staph. epi.) specified additional criteria required to make the diagnosis if the culture yielded CONS/Staph. epi. Of these additional criteria, two papers required concomitant abnormalities of the white blood cell count or CRP, one paper required positive cultures from two separate sites (usually drawn simultaneously), and one required culture to be positive within 48 h.
4. Septic shock/severe sepsis

Two papers specified additional criteria defining either septic shock or severe sepsis. The paper defining septic shock specified it as meeting the criteria for sepsis, plus blood pressure below the fifth centile for age requiring fluid resuscitation or inotropic support. The paper defining severe sepsis specified it as meeting the criteria for sepsis plus objective evidence of organ dysfunction.

## Discussion

There is considerable heterogeneity in the definitions of neonatal sepsis, both in the combinations of primary criteria used to define sepsis and in the specific secondary criteria in each category. This is huge problem for neonatology resulting in studies with most definitions not validated, sensitive or specific and using subjective criteria so not comparable or generalisable. Most notably, there was a reliance on microbiological culture for definitive diagnosis of sepsis in 85% of studies. This suggests that most of these definitions actually aim to describe ‘bacteraemia’ rather than ‘sepsis’. This is at variance with the adult Sepsis-3 guidelines^9,34^ and in contrast with the recent definitions of adult and paediatric sepsis which involve an ‘inflammatory host responses with end organ impairment’ independent of microbial detection. In additon, clinical signs of neonatal sepsis and infection are notoriously unreliable for diagnosis and can only trigger evaluation and may not be part of an actual definition as they are not specific for sepsis in neonates.

Most definitions using microbiological culture as an essential component for the diagnosis of sepsis vary in the exact requisites for a positive culture. Most papers specified a culture site and of those that did, most specified blood as the only site while a minority included other possible sites (e.g. CSF). Of the 83 definitions that included culture as a criterion, only 12 outlined what pathogens were assumed to be pathogenic which is significant in the context of CONS and the possibility of culture contamination versus nosocomial infection. Commensals represent a diagnostic quandary for neonatal sepsis, and approaches to CoNS detected in culture varied widely. No paper mentioned decisions in cases of a polymicrobial culture.^115^

Most definitions included clinical signs as a component of the definition of sepsis; however, papers varied in the precise definitions of certain signs and the overall list of signs. Certain signs predominate in frequency (Table 3) such as systemic signs, including lethargy, feeding intolerance and temperature instability, and cardiorespiratory signs including hypotension and respiratory distress. Two of the most frequent signs listed—hypotension and respiratory distress—as well as the platelet count are parameters of organ failure measured in the SOFA score in the Sepsis-3 guidelines^9^ and are identical to the three used for nSOFA score.^116^

Severe sepsis is now widely regarded to be an obsolete characterisation of the syndrome; however, it is notable that the study that defined severe sepsis characterised it as the presence of organ dysfunction, which is the key feature of the Sepsis-3 definition of sepsis in adults. Septic shock is a useful clinical distinction in adults and neonates, but one definition specifically labels septic shock and other definitions include instead low blood pressure in the definition of sepsis.^9^

A wide range of laboratory investigations appeared in the results, 36 definitions mentioned abnormalities of the white cell count—or related measures like the neutrophil count, immature neutrophil count, and immature:total neutrophil ratio and inflammation as marked by CRP or ESR. While radiological signs of infection were classed as a primary criterion, this in effect meant signs of pneumonia on chest X-ray, given that this was the only radiological investigation cited by any of the studies. Risk factors for infection were cited by several studies, which included a comprehensive range of risk factors such as chorioamnionitis, prolonged rupture of membranes, or foul-smelling amniotic fluid. There was variability in the timeframes defined for early-onset neonatal sepsis (< 2, < 3, < 5 days) with better agreement on the definition of late-onset sepsis.

Definitions of neonatal sepsis used in randomised clinical trials demonstrate considerable variability which means that comparison of studies and outcomes is very challenging.^117^ The major neonatal organisations such as National Institute of Child Health and Human Development (NICHD) Neonatal Research Network, Centers for Disease Control and Prevention (CDC), Nosocomial infection surveillance system for preterm infants on neonatology departments (NEO-KISS), Vermont Oxford Network, and the Canadian Neonatal Network also differ considerably in defining early and late-onset sepsis, the requirement for antibiotic use or clinical signs, and in the rules for including CoNS as pathogens.^34^

A concentration on microbiological culture persists in contrast to definitions used for children and adults with less inclusion of multiorgan dysfunction. The next steps in this process include a Delphi process including stakeholders internationally to develop a consensus definition with regular updates.^118,119^ The involvement of families is crucial in this process to ensure a definition that can be translated across healthcare professionals and families.^119,120^ Definitions of neonatal sepsis require standardisation for accurate diagnoses, epidemiological studies and data synthesis. Any sepsis definition that includes organ dysfunction first requires definition of normal organ function in the vulnerable preterm population which is still enormous challenge for the neonatologists internationally. Additional collection and analysis of clinical data of the physiology of the extreme preterm is necessary to define hypotension, thrombocytopenia, renal and liver dysfunction before including ‘end organ impairment’ as a critical factor for defining neonatal sepsis.

## Supplementary information

Appendix
