# Correction: Neonatal sepsis definitions from randomised clinical trials

**DOI:** 10.1038/s41390-024-03416-9

**Published:** 2024-07-29

**Authors:** Rían Hayes, Jack Hartnett, Gergana Semova, Cian Murray, Katherine Murphy, Leah Carroll, Helena Plapp, Louise Hession, Jonathan O’Toole, Danielle McCollum, Edna Roche, Elinor Jenkins, David Mockler, Tim Hurley, Matthew McGovern, John Allen, Judith Meehan, Frans B. Plötz, Tobias Strunk, Willem P. de Boode, Richard Polin, James L. Wynn, Marina Degtyareva, Helmut Küster, Jan Janota, Eric Giannoni, Luregn J. Schlapbach, Fleur M. Keij, Irwin K. M. Reiss, Joseph Bliss, Joyce M. Koenig, Mark A. Turner, Christopher Gale, Eleanor J. Molloy, On behalf of the Infection, Inflammation, Immunology and Immunisation (I4) section of the European Society for Paediatric Research (ESPR)

**Affiliations:** 1 Discipline of Paediatrics, Trinity College Dublin, the University of Dublin & Children’s Hospital Ireland (CHI) at Tallaght, Dublin, Ireland; 2 John Stearne Medical Library, Trinity College Dublin, St. James’ Hospital, Dublin, Ireland; 3 Trinity Translational Medicine Institute, St James Hospital, Dublin, Ireland; 4 Trinity Research in Childhood Centre (TRiCC), Trinity College Dublin, Dublin, Ireland; 5 Department of Paediatrics, Tergooi Hospital, Blaricum, The Netherlands; 6 Department of Paediatrics, Amsterdam UMC, University of Amsterdam, Emma Children’s Hospital, Amsterdam, The Netherlands; 7 Neonatal Health and Development, Telethon Kids Institute, Perth, WA Australia; 8 Neonatal Directorate, King Edward Memorial Hospital for Women, Perth, WA Australia; 9 Radboud Institute for Health Sciences, Department of Neonatology, Radboud University Medical Center, Amalia Children’s Hospital, Nijmegen, The Netherlands; 10 Division of Neonatal-Perinatal Medicine, Department of Pediatrics, Columbia University Medical Center, New York City, NY USA; 11 Department of Pediatrics, University of Florida, Gainesville, FL USA; 12 Department of Pathology, Immunology, and Laboratory Medicine, University of Florida, Gainesville, FL USA; 13 Department of Neonatology, Pirogov Russian National Research Medical University, Moscow, Russia; 14 Neonatology, Clinic for Paediatric Cardiology, Intensive Care and Neonatology, University Medical Centre Göttingen, Göttingen, Germany; 15 Neonatal Unit, Department of Obstetrics and Gynecology, Motol University Hospital and Second Faculty of Medicine, Prague, Czech Republic; 16 Institute of Pathological Physiology, First Faculty of Medicine, Charles University, Prague, Czech Republic; 17 Clinic of Neonatology, Department Mother-Woman-Child, Lausanne University Hospital and University of Lausanne, Lausanne, Switzerland; 18 Paediatric Critical Care Research Group, Child Health Research Centre, University of Queensland, Brisbane, QLD Australia; 19 Paediatric Intensive Care Unit, Queensland Children’s Hospital, Brisbane, QLD Australia; 20 Department of Pediatrics, Bern University Hospital, University of Bern, Bern, Switzerland; 21 Department of Pediatrics, Division of Neonatology, Erasmus MC-Sophia Children’s Hospital, Rotterdam, The Netherlands; 22 Department of Pediatrics, Women & Infants Hospital of Rhode Island, Alpert Medical School of Brown University, Providence, RI USA; 23 Division of Neonatology, Saint Louis University, Edward Doisy Research Center, St. Louis, MO USA; 24 Institute of Translational Medicine, University of Liverpool, Centre for Women’s Health Research, Liverpool Women’s Hospital, Liverpool, UK; 25 Neonatal Medicine, School of Public Health, Faculty of Medicine, Chelsea and Westminster campus, Imperial College London, London, UK; 26 Paediatrics, Coombe Women’s and Infant’s University Hospital, Dublin, Ireland; 27 Neonatology, CHI at Crumlin, Dublin, Ireland

Correction to: *Pediatric Research* 10.1038/s41390-021-01749-3, published online 06 November 2021

Appendix 1, which is mentioned in the text, was unfortunately missing from the original version and it has now been added. The original article has been corrected.

## Supplementary information

Appendix 1

